# Acute Liver Failure From Sickle Cell Hepatopathy Treated With Exchange Transfusion

**DOI:** 10.7759/cureus.15334

**Published:** 2021-05-30

**Authors:** Ahmad N Kassem, Changsu Park, Aarthi Rajkumar

**Affiliations:** 1 Internal Medicine, MetroHealth Medical Center, Cleveland, USA

**Keywords:** sickle cell crisis, acute liver failure, sickle cell hepatopathy, red blood cell exchange, exchange transfusion

## Abstract

Sickle cell disease (SCD) is a qualitative hemoglobinopathy that can cause widespread sickling and vaso-occlusive events in all organ systems. Sickle cell hepatopathy is an umbrella term for various acute and chronic pathologies of the liver as a result of sickling in SCD patients. We present below the case of a 49-year-old woman who had an acute liver failure in the setting of a hepatic crisis with recovery after exchange transfusion. Hepatic involvement in SCD may be life-threatening. Understanding the etiology and severity of hepatic involvement by sickling is necessary for appropriate treatment.

## Introduction

Sickle cell disease (SCD) is a qualitative hemoglobinopathy that can cause widespread sickling and vaso-occlusive events in all organ systems [1]. Sickle cell hepatopathy is a term used for a myriad of acute and chronic pathologies of the liver as a result of sickling in SCD patients. We present herein the case of a patient who developed acute liver failure in the setting of a sickle cell crisis with progressive improvement after blood exchange transfusion.

## Case presentation

A 49-year-old Hispanic African American female was presented to the emergency department with diffuse pains involving the abdomen, chest, and limbs of one-day duration that resemble the symptoms of her previous pain crises. She was known to have HbSS SCD with chronic anemia and complicated by end-stage renal disease (ESRD) requiring hemodialysis. She is a post-cholecystectomy patient. She did not use tobacco, alcohol, or recreational drugs. Her home medications included metoprolol, mirtazapine, omeprazole, cinacalcet, aspirin, calcitriol, calcium acetate phosphate binder, epoetin alpha injections, folic acid, and multivitamins. 

The patient’s review of systems was otherwise negative. On presentation, she had a blood pressure of 143/113 mmHg, a temperature of 36.1 °C, a heart rate of 101 beats per minute, and a SpO2 of 98% on room air. She was in mild distress and her abdomen was soft and diffusely tender. Besides the arteriovenous fistula in her left arm with a palpable thrill, her examination was otherwise unremarkable.

Her lab findings showed a white blood cell (WBC) count of 11.3 k/µL, a hemoglobin (Hb) level of 9.6 g/dL (close to baseline), and a platelet count of 166 k/µL. Her reticulocyte count was 0.21 M/µL at 7.3%. Na^+^ 137 mm/L, K^+^ 3.9 mmol/L, $HCO_{_{3}^{}}^{-}$ 30 mmol/L, Cl^−^ 94 mmol/L, BUN 12 mg/dL, creatinine 2.67 mg/dL, Ca^2+^ 8.9 mmol/L, Mg^2+^ 2.1 mg/dL, lactic acid 1.9 mmol/L, albumin 4g/dL with a total protein of 6.9 g/dL, direct/total bilirubin 0.7/2.7 mg/dL, alkaline phosphatase 670 IU/L (variable chronic elevation), alanine transaminase (ALT) 17 IU/L, and aspartate transaminase (AST) 57 IU/L. She was admitted and treated with opiates for pain control. Intravenous (IV) fluids were held given her ESRD awaiting hemodialysis.

**Figure 1:**
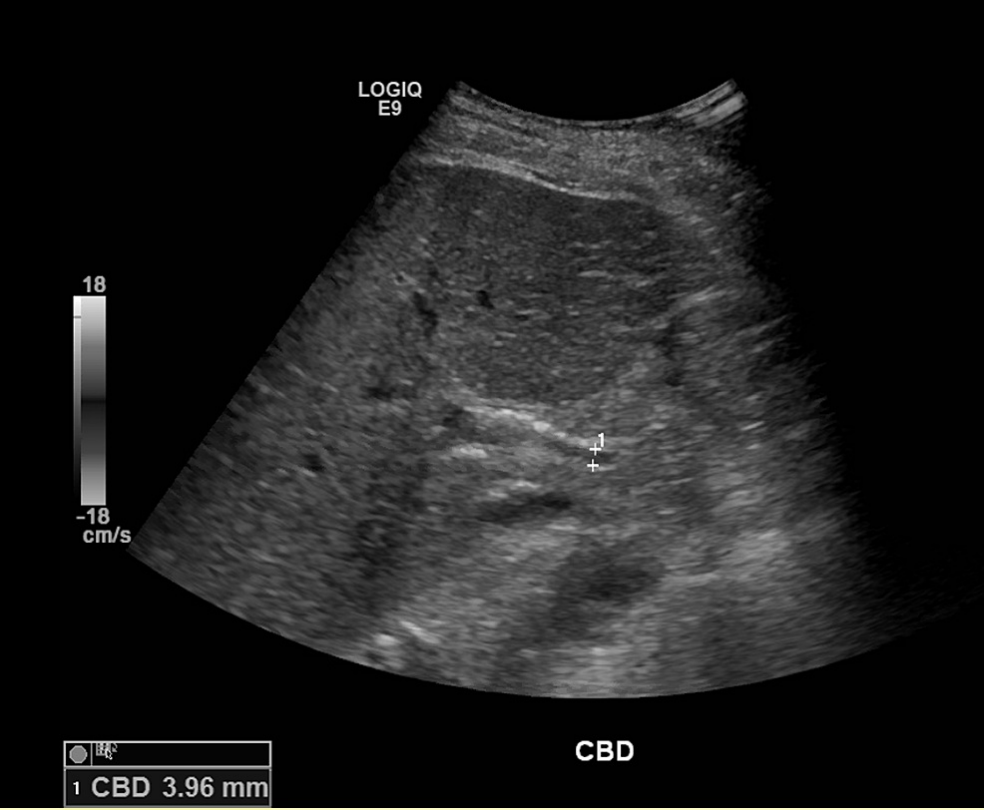
Liver ultrasound showing increased echogenicity of the hepatic parenchyma with no common bile duct dilation. CBD: common bile duct.

**Figure 2:**
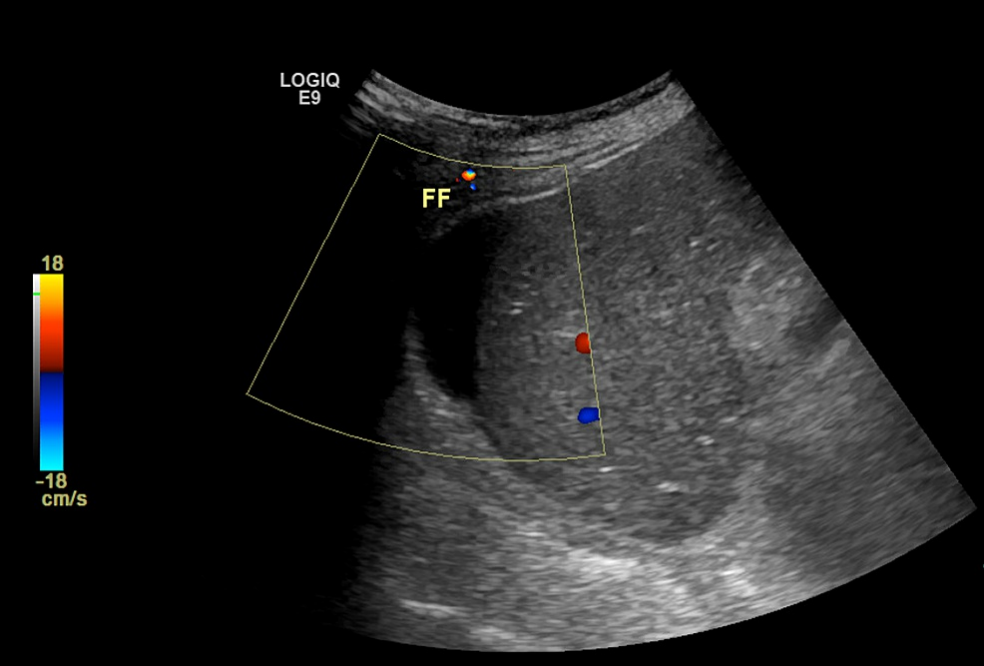
Status post cholecystectomy with a trace amount of free fluid in the gallbladder fossa. FF: free fluid.

**Figure 3:**
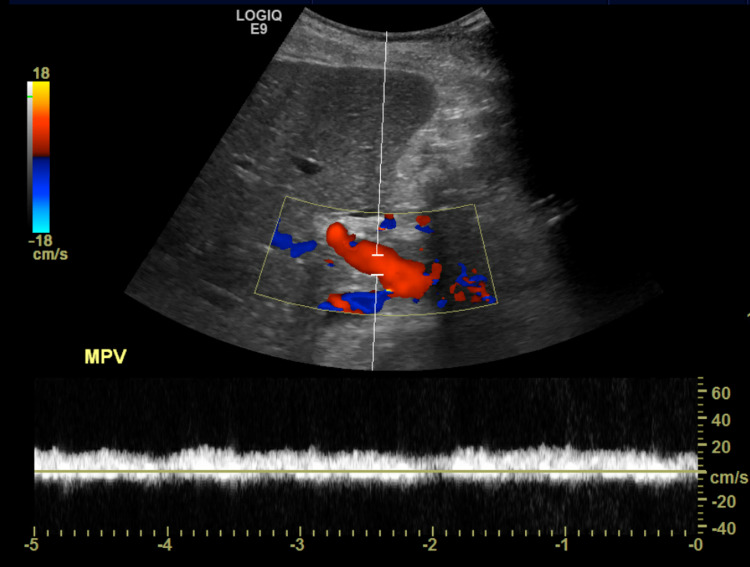
Doppler showing normal flow in the main portal vein. MPV: main portal vein.

On her third day in the hospital, the patient was noted to be lethargic. Her blood glucose was too low to measure on a glucometer. She was given IV dextrose with an incomplete improvement of her mentation. Her blood glucose dropped again overnight with a measured blood glucose of 42 g/dL. She was treated with dextrose and transferred to the medical intensive care unit (ICU) in an obtunded state. She required 2 l/min O_2_ via nasal cannula to maintain a SpO2>90%. Her examination at that point was remarkable for worsening conjunctival icterus, palpable hepatomegaly, and diffuse abdominal tenderness. Her lab results showed dramatic new derangements with a lactic acid of 12.5 mmol/L, direct/total bilirubin of 15.6/22.5 mg/dL, alkaline phosphatase 496 IU/L, ALT 571 IU/L, and AST 1440 IU/L. Her prothrombin time was 60.9 seconds corresponding with an INR of 5.48. Activated prothrombin time was slightly prolonged at 43 seconds. An ammonia level was measured at 37 umol/L. A random cortisol level was appropriately elevated at 59 µg/dl. A continuous dextrose infusion was initiated. Viral hepatitis serologies were negative. Liver ultrasound with doppler was done showing trace fluid in the gallbladder fossa with increased echogenicity of the hepatic parenchyma with normal hepatic and portal vascular circulation flow (Figures 1-3). The craniocaudal length of the liver was 17 cm.

The patient was hemodynamically stable throughout her course. Empirical broad-spectrum antibiotic coverage was initiated with cultures eventually negative. She had a blood exchange transfusion on her first day in the ICU. Her course over the next days was marked by a progressive, yet significant improvement of her mental status as she became easier to arouse, then was able to answer basic questions and obey command with waxing and waning levels of cognition over 72 hours. Her mentation normalized and she was transferred back to the regular ward. Her labs continued to improve. 

Of note, her course was notable for acute thrombocytopenia with a nadir platelet count of 33 k/µL. Given the acuity of the platelet drop, with a concomitant right cephalic vein superficial thrombophlebitis, she was treated for heparin-induced thrombocytopenia with Argatroban for four days which was eventually stopped after the screen for antibodies to the platelet factor 4/heparin complex was negative.

She was discharged feeling well after 12 days in the hospital with plans for outpatient follow-up with hematology and her primary care provider.

## Discussion

Sickle cell hepatopathy is a term used to refer to a spectrum of liver pathologies with various etiologies. The sickling process can be associated with hepatic injuries in many ways, including gall stone disease, hypoxic liver injury, hepatic sequestration, venous outflow obstruction, viral hepatitis (especially in multi-transfused patients), hepatic crisis, and sickle cell intrahepatic cholestasis [2].

Acute liver failure is a rare and severe consequence of abrupt hepatocyte injury manifesting as rapid-onset elevation of aminotransferases, altered mentation, and disturbed coagulation [3]. Acute liver injury is generally thought to involve an acute hepatic insult with sustained hepatic function. Our patient was admitted with a sickle cell crisis and developed liver failure first manifesting as encephalopathy and hypoglycemia. The rapidly progressive hepatic failure with encephalopathy, jaundice, and loss of synthetic function is rare; yet hepatic involvement in SCD is common and arguably underdiagnosed. Liver involvement in SCDs has been reported to be up to 39% in some studies with liver infarctions reported in as much as 34% of patients [4]. The histological manifestations of sickle cell hepatopathy may include obstruction, congestion, and dilation of sinusoids, ischemic necrosis, Kuppfer cell hyperplasia, erythrophagocytosis, and portal and perisinusoidal fibrosis following recurrent episodes of vaso-occlusions [5].

Our patient’s course was marked by rapid deterioration and a swift improvement after exchange transfusion. The relatively rapid improvement and full clinical recovery may suggest that our patient suffered from acute sickle cell hepatic crisis in which sinusoid obstruction results in hepatic ischemia and infarction which usually resolves within 3 to 14 days [6]. However, our patient’s presentation was complicated by coagulopathy and encephalopathy which is more consistent with acute intrahepatic cholestasis. Acute intrahepatic cholestasis is a diffuse sickling in the sinusoids leading to widespread ischemia with hypoxia that leads to the ballooning of hepatocytes and intracanalicular cholestasis [6]. A biopsy was not performed to determine the exact pathology. It is possible that our patient’s concomitant renal failure may have exacerbated the manifestations of her liver injury.

The evidence to support the benefit of exchange blood transfusion in sickle cell hepatopathy is scarce [7]. However, there is growing anecdotal evidence and a number of case reports suggesting improvement of acute intrahepatic cholestasis with exchange blood transfusion. A target of HbS levels of less than 20-30% has been suggested [8]. The decision to initiate and then stop exchange blood transfusion should be made on an individual basis. The decision to undergo exchange transfusion for our patient was made in the setting of her rapid clinical deterioration with subsequent improvement.

Worthy of discussion, in this case, is withholding intravenous fluids from this patient on admission as she is at risk of volume overload with her ESRD, and whether this decision was the correct one. Intravenous crystalloid infusion has been a mainstay treatment of sickle cell crisis. However, there is increasing evidence that driving sickle cell patients to hypervolemia is harmful [9] and intravenous fluid should be administered judiciously based on volume status [10]. Isotonic fluids which are most commonly used in volume resuscitation may be associated with increased red blood cell stiffness and an increased transit time in the microvasculature [11]. It remains unclear if a certain composition and rate of intravenous fluids in SCD are more liver-protective.

## Conclusions

This case sheds the light on the potentially life-threatening complications of intrahepatic sickling. Hepatic involvement in SCD is common with a wide spectrum of presentations. Our patient exhibited rapidly progressive liver failure with a quick improvement after exchange transfusion, favoring the benefit of this intervention. An astute assessment of the etiology and severity of hepatic involvement by sickling is necessary for appropriate treatment.
